# Predictive Factors for Two-Year Survival in Dogs with Hepatobiliary Diseases: Importance of Clinical and Laboratory Monitoring

**DOI:** 10.3390/ani13162677

**Published:** 2023-08-20

**Authors:** Sathidpak Nantasanti Assawarachan, Thodsapol Ongvisespaibool, Benjang Hakhen, Piyathip Chuchalermporn, Phudit Maneesaay, Naris Thengchaisri

**Affiliations:** 1 Department of Companion Animal Clinical Sciences, Faculty of Veterinary Medicine, Kasetsart University, Bangkok 10900, Thailand; sathidpak.n@ku.ac.th; 2 Endocrinology and Gastroenterology Unit, Kasetsart University Veterinary Teaching Hospital, Faculty of Veterinary Medicine, Kasetsart University, Bangkok 10900, Thailand; thodsapol.o@ku.th; 3 Surgical Unit, Kasetsart Veterinary Teaching Hospital, Faculty of Veterinary Medicine, Kasetsart University, Bangkok 10900, Thailand; benjang.h@ku.th; 4 Radiology Unit, Kasetsart University Veterinary Teaching Hospital, Faculty of Veterinary Medicine, Kasetsart University, Bangkok 10900, Thailand; piyathip.c@ku.th; 5 Department of Pathology, Faculty of Veterinary Medicine, Kasetsart University, Bangkok 10900, Thailand; fvetpdm@ku.ac.th

**Keywords:** idiopathic chronic hepatitis, liver fibrosis, vacuolar hepatopathy, prognosis, dog

## Abstract

**Simple Summary:**

Idiopathic chronic hepatitis, liver fibrosis, and vacuolar hepatopathy are the most common liver histological diagnoses of dogs with hepatobiliary diseases in Thailand. Clinical parameters and survival predictors that can forecast survival in dogs are poorly described. This research described clinical factors that help predict two-year survival in dogs after diagnosis of liver abnormalities following liver biopsy.

**Abstract:**

Long-term outcomes and survival predictors for different clinicopathologies (idiopathic chronic hepatitis, liver fibrosis, vacuolar hepatopathy) in dogs with hepatobiliary diseases are poorly described. In this study, ninety dogs were followed up for up to five years to investigate clinical factors that predict two-year survival in canine patients after liver biopsy. Univariate and multivariate analyses were performed based on clinical and laboratory data to determine the association between clinical and laboratory data and mortality rates. Overall, the one-, two-, and five-year mortality rates were 28.9%, 45.6%, and 78.9%, respectively. Univariate analysis indicated that male gender, ascites, elevated serum gamma-glutamyl transpeptidase (GGT), hypercholesterolemia, hypoalbuminemia, prolonged activated partial thromboplastin clotting time (aPTT), and prolonged thrombin clotting time (TT) were associated with an increased two-year mortality rate. Results from multivariate analysis demonstrated a significant association between male gender (*p* = 0.022), elevated serum GGT (*p* < 0.001), hypoalbuminemia (*p* < 0.001), and prolonged aPTT (*p* < 0.001) and an increased two-year mortality rate, regardless of the specific type of liver pathology. Elevated GGT was associated with the highest risk for increased two-year mortality (95% CI: hazard ratio 6.02–41.21). In conclusion, various clinical factors in dogs with liver diseases are useful for prognosis prediction.

## 1. Introduction

The prevalence of hepatobiliary diseases is increasing among dogs and cats globally, resulting in elevated levels of morbidity and mortality. Such diseases pose significant threats to the health and survival of small animals [1]. Accurate diagnosis of these conditions necessitates a combination of laboratory tests, imaging techniques, cytology, and histopathology [2,3,4]. Biochemical abnormalities were observed in canine patients with liver disease, including altered serum alanine aminotransferase (ALT), aspartate aminotransferase (AST), alkaline phosphatase (ALP), gamma-glutamyl transpeptidase (GGT), bile acids, bilirubin, cholesterol, triglyceride, albumin, and coagulation profiles [5]. While elevated liver enzymes serve as initial indicators of liver disease in dogs, their sensitivity in detecting all liver disorders remains relatively low, ranging from 60% to 76% [2,6]. ALT, primarily presented in the liver, is released into the bloodstream when hepatocyte membrane damage occurs, indicative of hepatocellular injury. Although ALT exhibited the highest sensitivity in detecting necrotic liver diseases, its efficacy in identifying neoplastic diseases was diminished, with sensitivity as low as 40% [2]. Moreover, its sensitivity for acute hepatitis in Labrador Retrievers was only 45% [7]. It is notable that chronic hepatitis C viral infections in humans might present with normal ALT activity [8]. ALP, another liver enzyme, is released in cholestatic disorders. Its sensitivity for detecting parenchymal liver disorders varies from 45% to 100% [2]. However, the sensitivity of ALP for acute hepatitis is merely 15% [6]. Dogs afflicted with liver disease demonstrated significantly elevated levels of liver enzymes, such as ALT and ALP, in comparison to a group of healthy canines [9]. Elevated liver enzymes are often used as an initial indicator for considering a liver biopsy [5]. Due to the limitations of ALT and ALP in early liver disease screening, their results must be interpreted in conjunction with signalment, medical history, physical findings, laboratory examinations, and imaging [2,6]. Although an ultrasonographic examination can detect some types of liver disease [9], liver biopsy remains essential for the diagnosis and classification of parenchymal liver diseases [3,10]. Given the importance of early liver disease detection for effective treatment, a non-invasive and more sensitive marker is needed to identify subclinical cases [1]. In dogs, cholestasis and hepatic insufficiency can lead to heightened levels of triglycerides and/or cholesterol [11]. Idiopathic chronic hepatitis is associated with hypertriglyceridemia and hypercholesterolemia. Elevated cholesterol levels in dogs with chronic hepatitis are significantly associated with serum liver enzymes and bile acid levels. Over half of the dogs with chronic hepatitis exhibited hypertriglyceridemia, hypercholesterolemia, or a combination of both [12]. Hyperlipidemia may be secondary to cholestasis, which is often found in canine chronic hepatitis [13]. In human patients, alterations in lipid profiles are dependent on the severity and type of liver disease [14]. 

Our previous study, following the World Small Animal Veterinary Association (WSAVA) guidelines, indicated that the three most common canine liver histological diagnoses in Thailand are chronic hepatitis (37.9%), liver fibrosis (19.5%), and vacuolar hepatopathy (10.3%) [15]. Idiopathic chronic hepatitis is characterized histologically by hepatocellular apoptosis or necrosis, inflammation of hepatic parenchyma, and fibrosis [16,17]. Vacuolar hepatopathy is likely a secondary reactive change in the liver to other diseases. It has been reported that approximately half of the dogs with vacuolar hepatopathy had no evidence of overt glucocorticoid exposure. Underlying primary disease may contribute to stress-induced hypercortisolemia resulting in the development of vacuolar hepatopathy. Fulminant hepatic dysfunction may develop secondary to severe vacuolar hepatopathy [18]. Understanding the relationship between biochemical abnormalities and hepatobiliary diseases is crucial for predicting the likelihood of these patients developing advanced liver disease. Examination of clinicobiochemical abnormalities can assist in formulating effective strategies for treating hepatobiliary diseases.

Clinical findings associated with poor prognosis in dogs with chronic hepatitis include the presence of ascites [19], hyperbilirubinemia [20], neutrophilia [21], and prolongation in PT [22]. Other important prognostic indicators in dogs with primary liver diseases are hypoalbuminemia, leukogram left shift [16], and a high systemic inflammatory response score [23]. However, the predictive factors of two-year survival in canine patients with liver diseases have not been fully described. It is not known whether different liver pathologies, such as idiopathic chronic hepatitis, liver fibrosis, and vacuolar hepatopathy, can affect the survival of canine patients. Moreover, the biochemical abnormalities that indicate the presence of liver pathologies may be useful for the prediction of early death in canine patients with liver disease. Additional investigation is needed to determine the specific clinical factors and biochemical abnormalities that are not only important for monitoring the progression of liver disease but are also able to provide a prognosis for these patients.

The purpose of the present study was to determine the clinical parameters and types of histological diagnoses (idiopathic chronic hepatitis, liver fibrosis, and vacuolar hepatopathy) that can predict two-year survival in dogs after diagnosis of liver abnormalities following liver biopsy.

## 2. Materials and Methods

A prospective study on client-owned dogs with a histological diagnosis of idiopathic chronic hepatitis, liver fibrosis, and vacuolar hepatopathy was conducted at the Kasetsart University Veterinary Teaching Hospital from January 2015 to December 2019. The use of animals in this study was approved by the Kasetsart University Institutional Animal Care and Use Committee (ACKU61-VET-012). Informed consent from each owner was obtained prior to the evaluation of dogs in the study. The dogs in the study were canine patients with either (1) persistent (more than 3 months) unexplained elevated liver enzymes (ALT and/or ALP) of greater than twice the upper limit of the reference range, or (2) less than twice the upper limit of the elevated liver enzymes but with abnormal ultrasonographic liver parenchymal appearance (i.e., irregular hypoechoic nodules, heterogenous liver echotexture). Dogs with infectious, copper-associated, toxic/drug-induced chronic hepatitis, and dogs with evidence of glucocorticoid exposure, hyperadrenocorticism, adrenomegaly, neoplastic diseases, and other concurrent diseases were excluded. Among the 90 dogs enrolled in the study, there were 25 mixed-breed dogs (27.78%), 17 Shih Tzu (18.89%), 11 Poodles (12.22%), 8 Chihuahuas (8.89%), 5 Siberian Huskies (5.56%), 4 West Highland White Terriers (4.44%), 3 Beagles (3.33%), 2 Golden Retrievers (2.22%), 2 Jack Russells (2.22%), 2 Labrador Retrievers (2.22%), 1 American Pitbull (1.11%), 1 Thai Bangkaew (1.11%), 1 Cocker Spaniel (1.11%), 1 Lhasa Apso (1.11%), 1 Maltese (1.11%), 1 Miniature Pinscher (1.11%), 1 Pekingese (1.11%), and 1 Pomeranian (1.11%). Data concerning signalment, medical history, clinical and laboratory findings, biopsy collection method, treatment, and follow-up care were obtained from medical records. Patients were followed up after liver biopsy for five years or until death from liver-related causes. Dogs with a body weight of 0 to 12 kg, 12.01 to 24 kg, and >24.01 kg were described as small, medium, and large breeds, respectively.

Blood samples were collected within one week (for punch biopsy) or on the same day (for needle biopsy) as the liver biopsy. The samples were taken from a cephalic or saphenous vein to assess complete blood count (CBC) and blood chemistry using a Sysmex XN-1000TM Hematology Analyzer (Sysmex, Mundelein, IL, USA) and an IL Lab 650 chemistry system (Diamond Diagnostics, Holliston, MA, USA), respectively. Prior to collecting blood samples, dogs were fasted for at least 12 h. Tests included blood urea nitrogen (BUN), creatinine, ALT, AST, ALP, GGT, total bilirubin, cholesterol, triglyceride, total protein, and albumin. Automated prothrombin time (PT), activated partial thromboplastin time (aPTT), and thrombin time (TT) were assessed with a coagulation analyzer (Amax Destiny Plus, Trinity Biotech) using a commercial reagent (Triniclot PT Excel S, Triniclot Automated APTT, Trinicot TT, Tcoag) according to the manufacturer’s instructions. Laboratory-specific reference intervals were used for PT, APTT, and TT in dogs. 

At the time of the original diagnosis, liver biopsies were obtained from animals under general anesthesia using 14-gauge automatic spring-loaded needles (Argon, Frisco, TX, USA) with ultrasound (GE, Chicago, IL, USA) guidance, or using a biopsy punch with exploratory celiotomy. At least three pieces of tissue were collected from multiple liver lobes to accurately diagnose liver disease [24]. A Thai board-certified pathologist with expertise in histological evaluation of liver diseases performed the histological diagnoses based on WSAVA guidelines [25].

Dogs diagnosed with idiopathic chronic hepatitis (55/90) were treated with oral prednisolone (1–1.5 mg/kg/day, Charoenbhaesaj Lab Co., Ltd., Bangkok, Thailand) for at least two months. Subsequently, the prednisolone dose was reduced by 25 percent to 50 percent every two to four weeks until it reached 0.25 mg/kg/every other day and then treatment ceased. Liver biopsy was not repeated in all dogs. The diets of the dogs were also supplemented with hepatoprotectants (silymarin (Samarin^®^, Berlin Pharmaceutical Industry Co., Ltd., Bangkok, Thailand) or a combination of S-adenosylmethionine, silymarin, vitamin C, and vitamin E (Samylin^®^, Vetplus, Lancashire, UK)) and ursodeoxycholic acid (Ursolin^®^, Berlin Pharmaceutical Industry Co., Ltd., Bangkok, Thailand). Diuretics (spironolactone (Hyles^®^, Berlin Pharmaceutical Industry Co., Ltd., Bangkok, Thailand) and low-protein diets (hepatic^®^, Royal Canin, Bangkok, Thailand or l/d^®^ Hill’s Pet Nutrition, Bangkok, Thailand) were prescribed in idiopathic chronic hepatitis dogs with clinical signs of portal hypertension (ascites; *n* = 4). The dogs with liver fibrosis and vacuolar hepatopathy were treated with palliative hepatoprotectants and ursodeoxycholic acid. 

Descriptive statistics are reported as the mean ± standard deviation. A Student’s *t*-test was used to compare parametric data including dog characteristics and biochemical values between dogs that died within two years and those that were alive for two years or longer. Statistically significance was set as *p* < 0.05. Univariate logistic regression was used to compare the association of each parameter between groups. For the multivariate analyses, all variables that were explored in the univariate analyses were considered using a stepwise approach. Significance thresholds of 0.05 and 0.1 were used to determine the qualification of data for entry into and deletion from the model, respectively. The Kaplan–Meier method was used to estimate survival rates. Statistical analyses were performed using STATA12.1 (StataCorp, College Station, TX, USA).

## 3. Results

**Figure 1:**
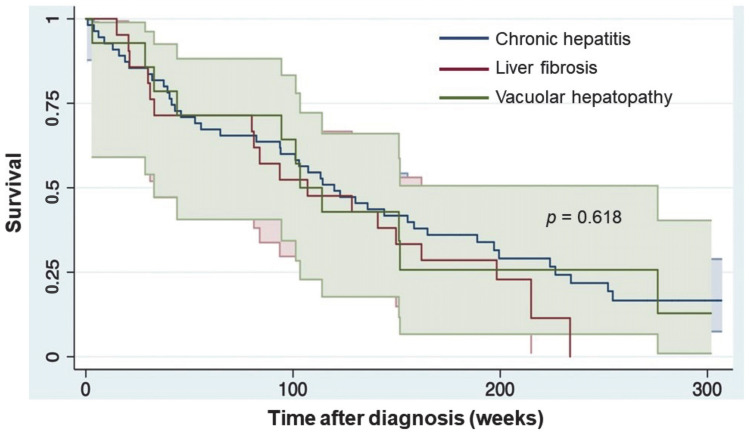
Kaplan–Meier survival plot of five-year survival from the day of diagnosis in dogs with liver diseases over a 300-week period.

A total of 90 canine patients with hepatic parenchymal lesions were included in this study. The histological diagnoses identified in dogs were idiopathic chronic hepatitis (61%, 55/90 dogs), liver fibrosis (23%, 21/90 dogs), and vacuolar hepatopathy (16%, 14/90 dogs). The patients’ baseline characteristics are presented in Table 1. Subjects in the study group consisted of 43 male (47.8%) and 47 female (52.2%) dogs with an average age of 10.2 ± 0.5 years. The mean age of dogs that survived beyond two years (10.6 ± 3.4 years) was marginally higher compared to those that succumbed within two years (9.9 ± 2.9 years, *p* = 0.358). No significant differences were observed in sex between dogs that had died or remained alive within two years. Similarly, the size categorization of dogs (small, medium, large) did not exert a substantial influence on survival outcomes (*p* = 0.541). Biochemical analyses revealed noteworthy insights, with dogs experiencing shorter survival periods exhibiting markedly elevated GGT levels (26 ± 57 U/L) in contrast to longer-surviving counterparts (6 ± 4 U/L, *p* = 0.016) (Table 1). Additionally, triglyceride levels were notably lower in the group with limited survival (139 ± 101 mg/dL) compared to those that survived beyond two years (259 ± 346 mg/dL, *p* = 0.046). The aPTT was also significantly higher (*p* = 0.006) in the dogs that died within two years. Conversely, no substantial statistical differences (*p* > 0.05) were observed in other biochemical markers, including ALT, AST, ALP, albumin, BUN, bilirubin, creatinine, cholesterol, PT, and TT. Clinical indicators such as hepatic encephalopathy, ascites, hypoalbuminemia, and hyperbilirubinemia displayed limited associations with the survival of dogs within two years (Table 1). Overall, the one-, two-, and five-year mortality were 28.9%, 45.6%, and 78.9%, respectively. The analysis of the Kaplan–Meier survival curve did not show any significant correlation between the histopathological classification of hepatobiliary diseases and survival (Figure 1). 

The results of univariate logistic regression analysis, conducted to assess the ability of clinical features to predict mortality at two years, are shown in Table 2. Male gender (*p* < 0.064), ascites at the time of biopsy (*p* < 0.001), elevated serum GGT greater than 25 U/L (*p* < 0.000), hypercholesterolemia (cholesterol > 335 mg/dL) (*p* < 0.078), hypoalbuminemia (albumin < 2.3 mg/dL) (*p* < 0.002), prolonged aPTT greater than 15 s (*p* = 0.000), and prolonged TT greater than 25 s (*p* < 0.071) were associated with increased mortality. Among the factors examined, fibrosis lesion, hyperbilirubinemia, hypertriglyceridemia, and portal hypertension were found to be non-significant (*p* > 0.100) in terms of their impact on the two-year survival of dogs with liver abnormalities and were not included in the multivariate analysis. In the multivariate analysis (Table 3), it was observed that male gender (hazard ratio 2.2, 95% CI 1.12–4.34, *p* < 0.022), elevated serum GGT greater than 25 U/L (hazard ratio 15.76, 95% CI 6.02–41.21, *p* = 0.000), hypoalbuminemia (hazard ratio 14.84, 95% CI 3.73–58.93, *p* = 0.000), and prolonged aPTT (hazard ratio 3.84, 95% CI 1.92–7.67, *p* = 0.000) were significantly associated with an increased risk of death within two years. The highest hazard risk was associated with elevated serum GGT greater than 25 U/L. 

## 4. Discussion

The escalating prevalence of hepatobiliary diseases in dogs and cats is resulting in increased morbidity and mortality rates, posing significant threats to the well-being and survival of small animals. Although initial indicators of liver diseases often involve elevated liver enzymes, their sensitivity remains limited, necessitating more precise markers. In the present study, clinical factors and histological diagnoses that predict two-year survival in canine patients with idiopathic chronic hepatitis, liver fibrosis, and vacuolar hepatopathy were evaluated. Male gender, ascites, elevated serum GGT, hypercholesterolemia, hypoalbuminemia, prolonged aPTT, and prolonged TT were associated with an increased two-year mortality rate using univariate analysis. In the multivariate analysis, male gender, elevated serum GGT (>25 U/L), hypoalbuminemia (<2.3 g/dL), and prolonged aPTT (>15 s) were significantly associated with an increased two-year mortality rate, irrespective of the specific type of liver pathology. Elevated GGT was associated with the highest risk for mortality within two years, with a hazard ratio of 6.02–41.21. These findings suggest that various clinical factors contribute to the prognosis of dogs with liver diseases.

Canine patients with the three most common liver histological diagnoses in Thailand were included in this study, namely idiopathic chronic hepatitis, liver fibrosis, and vacuolar hepatopathy [15]. Based on the Kaplan–Meier survival curve, the survival times in dogs with idiopathic chronic hepatitis were similar to dogs with histologic evidence of only vacuolar or fibrotic degenerative changes on hepatic biopsy. This indicates that the different types of liver histopathology did not have a significant impact on the survival rate of dogs. It is possible that the dogs with idiopathic chronic hepatitis in our study received appropriate treatment using prednisolone, an immunosuppressive drug that has been shown to reduce hepatic inflammation and limit progression to cirrhosis [26,27,28]. Corticosteroid treatment has also been shown to significantly improve the survival time of dogs with chronic hepatitis [29]. In studies of dogs with chronic hepatitis, where the treatment regimen was not standardized, median survival times were 189 days [30], 374 days [22], and 549 days (18.3 months) [16]. In contrast, when dogs with chronic hepatitis were treated with prednisolone and/or azathioprine, survival lengths of 913 days [31] and 1715 days [28] were reported. The median survival time of dogs in our study was 798 days (114 weeks), consistent with the need for immunosuppressive treatment of idiopathic chronic hepatitis to limit disease progression and increase the survival rate. 

Univariate logistic regression analysis from canine patients with biopsy-proven liver diseases revealed clinical parameters that may lead to early death in dogs with liver diseases. Our analysis indicates that male gender, the presence of ascites, elevated serum GGT level, hypercholesterolemia, hypoalbuminemia, prolonged aPTT, and prolonged TT are associated with reduced two-year survival. Ascites due to reduced oncotic pressure only occur when albumin concentrations are below 1.5 mg/dL [32]. The presence of ascites in dogs from this study group was likely caused by hepatic portal hypertension rather than hypoalbuminemia, as all dogs with ascites had serum albumin levels greater than 2.3 mg/dL. Signs of hepatic encephalopathy, gastrointestinal bleeding, and ascites are often observed in dogs with portal hypertension caused by prehepatic or hepatic lesions [33]. Common causes of hepatic portal hypertension are conditions that affect the entire liver such as chronic hepatitis, fibrosis, and cirrhosis [32,33]. Previous studies have demonstrated that ascites is a negative prognostic indicator in canine idiopathic chronic hepatitis [19], in addition to other factors such as the presence of cirrhosis [16,26]. 

Hypercholesterolemia was found in our analysis to be related to shorter survival times in dogs with parenchymal liver lesions. A previous study identified a strong correlation between high levels of liver enzymes and hypercholesterolemia in dogs with idiopathic chronic hepatitis [34]. This suggests that the degree of hypercholesterolemia may be associated with the severity of liver injury and the impairment of bile flow. Additionally, a mild correlation has been observed between high levels of serum bile acids and hypercholesterolemia [34]. In contrast, hypercholesterolemia was associated with well-preserved hepatic function and decreased mortality in humans with cirrhosis [35]. Whether hypercholesterolemia is related to a negative or positive prognosis may depend on the stage of liver disease. The presence of hypercholesterolemia during early liver diseases may suggest a higher degree of liver injury and cholestasis, which is associated with greater disease severity. However, hypercholesterolemia in patients with end-stage liver diseases may indicate better liver function.

The results of our univariate analysis demonstrated that hypoalbuminemia was a prognostic factor for shortened survival time after the diagnosis of idiopathic chronic hepatitis, liver fibrosis, and vacuolar hepatopathy. A similar result was found in a retrospective study of dogs with primary hepatitis including acute, chronic, lobular dissecting, granulomatous, and eosinophilic hepatitis. This may be due to the relationship between hypoalbuminemia and decompensated liver function [16]. However, a study on Labrador Retrievers with chronic hepatitis did not reveal an association between survival time and hypoalbuminemia [22].

The factors that regulate procoagulation, anticoagulation, and fibrinolysis are synthesized by the liver [36]. A prolonged aPTT score indicates deficiencies of intrinsic (factors XII, XI, IX, and VIII) and common (factors I (fibrinogen), II (prothrombin), V, and X) pathways. The TT and fibrinogen concentration measure the conversion of fibrinogen to fibrin, which is sensitive to fibrinogen defects [37]. Our study demonstrated that prolonged aPTT and TT were associated with shorter survival time in dogs with liver disease. Nonetheless, TT is not commonly measured in small animal clinics, instead measurement of fibrinogen concentration is more common. Our results suggest further research is needed to determine the clinical significance of TT, as a marker for disease severity and prognosis in dogs with liver disease.

In the present study, using multivariate analysis, it was found that male gender, elevated serum GGT concentrations, hypoalbuminemia, and prolonged aPTT were independently associated with a significantly increased risk of death within two years. Our study is the first to report a relationship between shorter survival time and male dogs with parenchymal liver lesions. Consistent with our findings, in humans, men have higher rates of death from chronic liver disease and cirrhosis, including those caused by alcohol-related liver disease, compared to women [38]. Previously, it was reported that there is a link between the prolongation of PT [22] or prolonged PT and aPTT and shortened survival time in dogs with various primary hepatitis [16]. Our analysis indicates that elevated serum GGT concentrations are associated with the highest risk for shortened survival time. An elevation of GGT was reported as a negative prognostic indicator in dogs with hepatocellular carcinoma and biliary surgery [38,39]. However, we are the first to report a link between elevated GGT and the risk of death in dogs with parenchymal liver diseases. High GGT levels are also a predictor of mortality in humans with non-hepatic diseases such as cardiovascular diseases, chronic kidney disease, and neoplasia [40]. It is important to note that the elevations of common liver enzymes (ALT and ALP) or the presence of dyslipidemia do not necessarily indicate a higher risk of death in dogs due to liver disease. 

Hyperbilirubinemia was previously demonstrated to be an important negative prognostic indicator in dogs with primary hepatitis [16,20]. However, our study did not find a link between hyperbilirubinemia and shorter survival time. A similar finding was reported in a population of Labrador Retrievers with chronic hepatitis, in which total serum bilirubin did not correlate with prognosis [22]. Given these outcomes, it is worth considering the possibility that a larger enrollment of canine patients may be necessary to comprehensively evaluate the role of bilirubin as a prognostic factor for hepatobiliary diseases.

Caution should be used when interpreting results from dogs with different types of liver disease since prognosis depends on multiple variables. When interpreting the results of the present study, it is important to consider these limitations. First, the present study was conducted in Bangkok, Thailand, and environmental factors in different regions, genetic backgrounds, and access to healthcare could influence the incidence or presentation of hepatic diseases. Second, there is no age- and sex-matched control group in this study. Another limitation is that the study only included dogs with specific types of liver diseases: idiopathic chronic hepatitis, liver fibrosis, and vacuolar hepatopathy. Therefore, the results of the study may not apply to other types of liver diseases, such as liver and gall bladder cancer and congenital liver diseases such as portosystemic shunt, copper-associated chronic hepatitis, or metabolic liver diseases. 

## 5. Conclusions

The clinical and histological factors that could be used to predict the two-year survival of dogs with liver disease were evaluated in the present study. Our findings suggested that male gender, elevated serum GGT, hypoalbuminemia, and prolonged aPTT are significantly associated with an increased two-year mortality rate, regardless of whether the morphologic lesions in the liver were idiopathic chronic hepatitis, liver fibrosis, or vacuolar hepatopathy. Elevated GGT levels were found to be the most significant risk factor for mortality in dogs with liver disease. The outcomes of the present study underscore the significance of these clinical and laboratory parameters in prognosticating canines with hepatic anomalies. By consolidating these impactful markers, this investigation provides an indispensable paradigm for veterinary clinicians and researchers to refine their prognostic assessments, fostering more precise treatment strategies and improved patient care in dogs with hepatobiliary diseases.

## Figures and Tables

**Table 1:** Baseline clinical and biochemical characteristics (mean ± SD) of dogs affected with liver diseases.

	Values	Death (within 2 Years)	Alive (More Than 2 Years)	*p* Value
*N*	90	41 (45.6%)	49 (54.4%)	
Age	10.2 ± 0.5	10.6 ± 3.4	9.9 ± 2.9	0.358
Sex				
Male	43 (47.8%)	23 (56.1%)	20 (40.8%)	
Female	47 (52.2%)	18 (43.9%)	29 (59.2%)	0.204
Size				
Small	55 (61.2%)	26 (63.4%)	29 (59.2%)	
Medium	22 (24.4%)	11 (26.8%)	11(22.4%)	
Large	13 (14.4%)	4 (9.8%)	9 (18.4%)	0.541
ALT, U/L	267 ± 538	342 ± 743	204 ± 259	0.228
AST, U/L	52 ± 91	65 ± 129	40 ± 29	0.201
ALP, U/L	875 ± 1579	1170 ± 2123	627 ± 852	0.104
GGT, U/L	15 ± 39	26 ± 57	6 ± 4	0.016
Albumin, g/dL	3.3 ± 0.5	3.2 ± 0.6	3.3 ± 0.3	0.102
BUN, mg/dL	16.8 ± 8.2	18 ± 8	16 ± 8	0.402
Bilirubin, mg/dL	0.5 ± 2.0	0.8 ± 2.9	0.2 ± 0.3	0.181
Creatinine, mg/dL	0.83 ± 0.23	0.82 ± 0.28	0.84 ± 0.17	0.720
Cholesterol, mg/dL	291 ± 182	322 ± 253	265 ± 84	0.151
Triglyceride, mg/dL	206 ± 272	139 ± 101	259 ± 346	0.046
Hepatic encephalopathy	1 (1.1%)	1 (2.0%)	0 (0.0%)	1.000
Ascites	4 (4.4%)	4 (9.8%)	0 (0.0%)	0.040
Hypoalbuminemia (<2.3 g/dL)	3 (3.3%)	3 (7.3%)	0 (0.0%)	0.091
Hyperbilirubinemia (>0.5 mg/dL)	9 (10.2%), *n* = 88	3 (7.3%), *n* = 41	6 (12.7%), *n* = 47	0.494
PT, second	5.5 ± 1.1	5.5 ± 1.3	5.4 ± 1.0	0.461
aPTT, second	14.0 ± 1.9	14.6 ± 2.0	13.5 ± 1.6	0.006
TT, second	24.0 ± 5.8	25.2 ± 7.3	23.0 ± 4.0	0.063
50% Survival, weeks	114	-	-	-
1-year mortality, %	26 (28.9%)	-	-	-
2-year mortality, %	41 (45.6%)	-	-	-
5-year mortality, %	71 (78.9%)	-	-	-

ALT, alanine aminotransferase; AST, aspartate aminotransferase; ALP, alkaline phosphatase; GGT, gamma-glutamyl transpeptidase; BUN, blood urea nitrogen; PT, prothrombin time; aPTT, activated partial thromboplastin time; TT, thrombin time.

**Table 2:** Unadjusted model for two-year survival predictors in dogs with liver diseases.

Parameters	Hazard Ratio (95% CI)	*p* Value
Ascites	6.5 (2.27–19.09)	0.001
Elevated GGT > 25 U/L	12.33 (5.28–28.82)	0.000
Fibrosis lesion	0.81 (0.42–1.55)	0.531
Hyperbilirubinemia	0.76 (0.23–2.47)	0.650
Hypercholesterolemia	1.85 (0.93–3.68)	0.078
Hypertriglyceridemia	0.96 (0.50–1.83)	0.907
Hypoalbuminemia	6.71 (2.01–22.37)	0.002
Male gender	1.79 (0.97–3.32)	0.064
Prolonged aPTT > 15 s	3.18 (1.67–6.08)	0.000
Prolonged TT > 25 s	1.78 (0.95–3.35)	0.071
Portal hypertension	0.86 (0.48–1.55)	0.626

**Table 3:** Adjusted model for two-year survival predictors of dogs with liver diseases.

Parameters	Hazard Ratio (95%CI)	*p* Value
Elevated GGT > 25 U/L	15.76 (6.02–41.21)	0.000
Hypoalbuminemia	14.84 (3.73–58.93)	0.000
Male gender	2.20 (1.12–4.34)	0.022
Prolonged aPTT > 15 s	3.84 (1.92–7.67)	0.000

## Data Availability

The datasets generated and/or analyzed during the current study are available from the corresponding author upon reasonable request.

## References

[1] Lidbury J.A., Suchodolski J.S. (2016). New advances in the diagnosis of canine and feline liver and pancreatic disease. Vet. J..

[2] Center S.A. (2007). Interpretation of liver enzymes. Vet. Clin. North Am. Small Anim. Pract..

[3] Alvarez L., Whittemore J. (2009). Liver enzyme elevations in dogs: Diagnostic approach. Compend. Contin. Educ. Vet..

[4] Favier R.P. (2009). Idiopathic hepatitis and cirrhosis in dogs. Vet. Clin. North Am. Small Anim. Pract..

[5] Chapman S.E., Hostutler R.A. (2015). A Laboratory Diagnostic Approach to Hepatobiliary Disease in Small Animals. Clin. Lab. Med..

[6] Sevelius E. (1995). Diagnosis and prognosis of chronic hepatitis and cirrhosis in dogs. J. Small Anim. Pract..

[7] Dirksen K., Burgener I.A., Rothuizen J., van den Ingh T.S.G.A.M., Penning L.C., Spee B.B., Fieten H. (2017). Sensitivity and specificity of plasma ALT, ALP, and bile acids for hepatitis in Labrador retrievers. J. Vet. Inter. Med..

[8] Hoofnagle J.H. (1997). Hepatitis C: The clinical spectrum of disease. Hepatology.

[9] Assawarachan S.N., Chuchalermporn P., Maneesaay P., Thengchaisri N. (2019). Evaluation of hepatobiliary ultrasound scores in healthy dogs and dogs with liver diseases. Vet. World.

[10] Rothuizen J., Desmet V.J., van den Ingh T.S.G.A.M., Twedt D.C., Bunch S.E., Washabau R.J., Rothuizen J., Bunch S.E., Charles J.A., Cullen J.M., Desmet V.J., Szatmari V., Twedt D.C., van den Ingh T.S.G.A.M., van Winkle T. (2009). Sampling and handling of liver tissue. WSAVA Standards for Clinical and Histological Diagnosis of Canine and Feline Liver Diseases.

[11] Xenoulis P.G., Steiner J.M. (2015). Canine hyperlipidaemia. J. Small Anim. Pract..

[12] Assawarachan S.N., Chuchalermporn P., Maneesaay P., Thengchaisri N. (2021). Changes in Serum Lipid Profiles among Canine Patients Suffering from Chronic Hepatitis. Vet. Sci..

[13] Boisclair J., Doré M., Beauchamp G., Chouinard L., Girard C. (2001). Characterization of the inflammatory infiltrate in canine chronic hepatitis. Vet. Pathol..

[14] Unger L.W., Forstner B., Schneglberger S., Muckenhuber M., Eigenbauer E., Scheiner B., Mandorfer M., Trauner M., Reiberger T. (2019). Patterns and prevalence of dyslipidemia in patients with different etiologies of chronic liver disease. Wien. Klin. Wochenschr..

[15] Assawarachan S.N., Maneesaay P., Thengchaisri N. (2020). A descriptive study of the histopathologic and biochemical liver test abnormalities in dogs with liver disease in Thailand. Can. J. Vet. Res..

[16] Poldervaart J.H., Favier R.P., Penning L.C., van den Ingh T.S., Rothuizen J. (2009). Primary hepatitis in dogs: A retrospective review (2002–2006). J. Vet. Intern. Med..

[17] Bexfield N. (2017). Canine idiopathic chronic hepatitis. Vet. Clin. North Am. Small Anim. Pract..

[18] Sepesy L.M., Center S.A., Randolph J.F., Warner K.L., Erb H.N. (2006). Vacuolar hepatopathy in dogs: 336 cases (1993–2005). J. Am. Vet. Med. Assoc..

[19] Raffan E., McCallum A., Scase T.J., Watson P.J. (2009). Ascites is a negative prognostic indicator in chronic hepatitis in dogs. J. Vet. Intern. Med..

[20] Gómez Selgas A., Bexfield N., Scase T.J., Holmes M.A., Watson P. (2014). Total serum bilirubin as a negative prognostic factor in idiopathic canine chronic hepatitis. J. Vet. Diagn. Invest..

[21] Breheny C.R., Handel I., Banner S., Milne E.M., Morrison L.R., Smith S.H., Kilpatrick S., Gow A., Mellanby R.J. (2020). Neutrophilia is associated with a poorer clinical outcome in dogs with chronic hepatitis. Vet. Rec..

[22] Shih J.L., Keating J.H., Freeman L.M., Webster C.R.L. (2007). Chronic hepatitis in Labrador Retrievers: Clinical presentation and prognostic factors. J. Vet. Intern. Med..

[23] Kilpatrick S., Dreistadt M., Frowde P., Powell R., Milne E., Smith S., Morrison L., Gow A.G., Handel I., Mellanby R.J. (2016). Presence of Systemic Inflammatory Response Syndrome Predicts a Poor Clinical Outcome in Dogs with a Primary Hepatitis. PLoS ONE.

[24] Kemp S.D., Zimmerman K.L., Panciera D.L., Monroe W.E., Leib M.S. (2015). Histopathologic variation between liver lobes in dogs. J. Vet. Intern. Med..

[25] Van den Ingh T.S.G.A.M., Van Winkle T., Cullen J.M., Charles J.A., Desmet V.J., Rothuizen J., Bunch S.E., Charles J.A., Cullen J.M., Desmet V.J., Szatmari V., Twedt D.C., van den Ingh T.S.G.A.M., van Winkle T. (2009). Morphological Classification of Parenchymal Disorders of the Canine and Feline Liver. WSAVA Standards for Clinical and Histological Diagnosis of Canine and Feline Liver Diseases.

[26] Favier R.P., Poldervaart J.H., van den Ingh T.S., Penning L.C., Rothuizen J. (2013). A retrospective study of oral prednisolone treatment in canine chronic hepatitis. Vet. Q..

[27] Webster C.R.L., Center S.A., Cullen J.M., Penninck D.G., Richter K.P., Twedt D.C., Watson P.J. (2019). ACVIM consensus statement on the diagnosis and treatment of chronic hepatitis in dogs. J. Vet. Intern. Med..

[28] Bayton W., Watson P.J., Bexfield N.H. (2020). Prednisolone therapy for chronic hepatitis in English springer spaniels: A prospective study of 12 cases. Vet. Rec..

[29] Strombeck D.R., Miller L.M., Harrold D. (1988). Effects of corticosteroid treatment on survival time in dogs with chronic hepatitis: 151 cases (1977–1985). J. Am. Vet. Med. Assoc..

[30] Bexfield N.H., Andres-Abdo C., Scase T.J., Constantino-Casas F., Watson P.J. (2011). Chronic hepatitis in the English springer spaniel: Clinical presentation, histological description and outcome. Vet. Rec..

[31] Kanemoto H., Sakai M., Sakamoto Y., Spee B., van den Ingh T.S.G.A.M., Schotanus B.A., Ohno K., Rothuizen J. (2013). American Cocker Spaniel chronic hepatitis in Japan. J. Vet. Intern. Med..

[32] Rothuizen J. (2009). Important clinical syndromes associated with liver disease. Vet. Clin. North Am. Small Anim. Pract..

[33] Bunch S.E., Johnson S.E., Cullen J.M. (2001). Idiopathic noncirrhotic portal hypertension in dogs: 33 cases (1982–1998). J. Am. Vet. Med. Assoc..

[34] Kaplan D.E., Serper M.A., Mehta R., Fox R., John B., Aytaman A., Baytarian M., Hunt K., Albrecht J., Njei B. (2019). VOCAL Study group. Effects of Hypercholesterolemia and Statin Exposure on Survival in a Large National Cohort of Patients with Cirrhosis. Gastroenterology.

[35] Webster C.R.L. (2017). Hemostatic Disorders Associated with Hepatobiliary Disease. Vet. Clin. North Am. Small Anim. Pract..

[36] Brooks M.B., Catalfamo J.L. (2013). Current diagnostic trends in coagulation disorders among dogs and cats. Vet. Clin. North Am. Small Anim. Pract..

[37] Guy J., Peters M.G. (2013). Liver disease in women: The influence of gender on epidemiology, natural history, and patient outcomes. Gastroenterol. Hepatol..

[38] Amsellem P.M., Seim H.B., MacPhail C.M., Bright R.M., Twedt D.C., Wrigley R.H., Monnet E. (2006). Long-term survival and risk factors associated with biliary surgery in dogs: 34 cases (1994–2004). J. Am. Vet. Med. Assoc..

[39] Moyer J., Lopez D.J., Balkman C.E., Sumner J.P. (2021). Factors associated with survival in dogs with a histopathological diagnosis of hepatocellular carcinoma: 94 cases (2007–2018). Open Vet. J..

[40] Brennan P.N., Dillon J.F., Tapper E.B. (2022). Gamma-Glutamyl Transferase (γ-GT)—An old dog with new tricks?. Liver Int..

